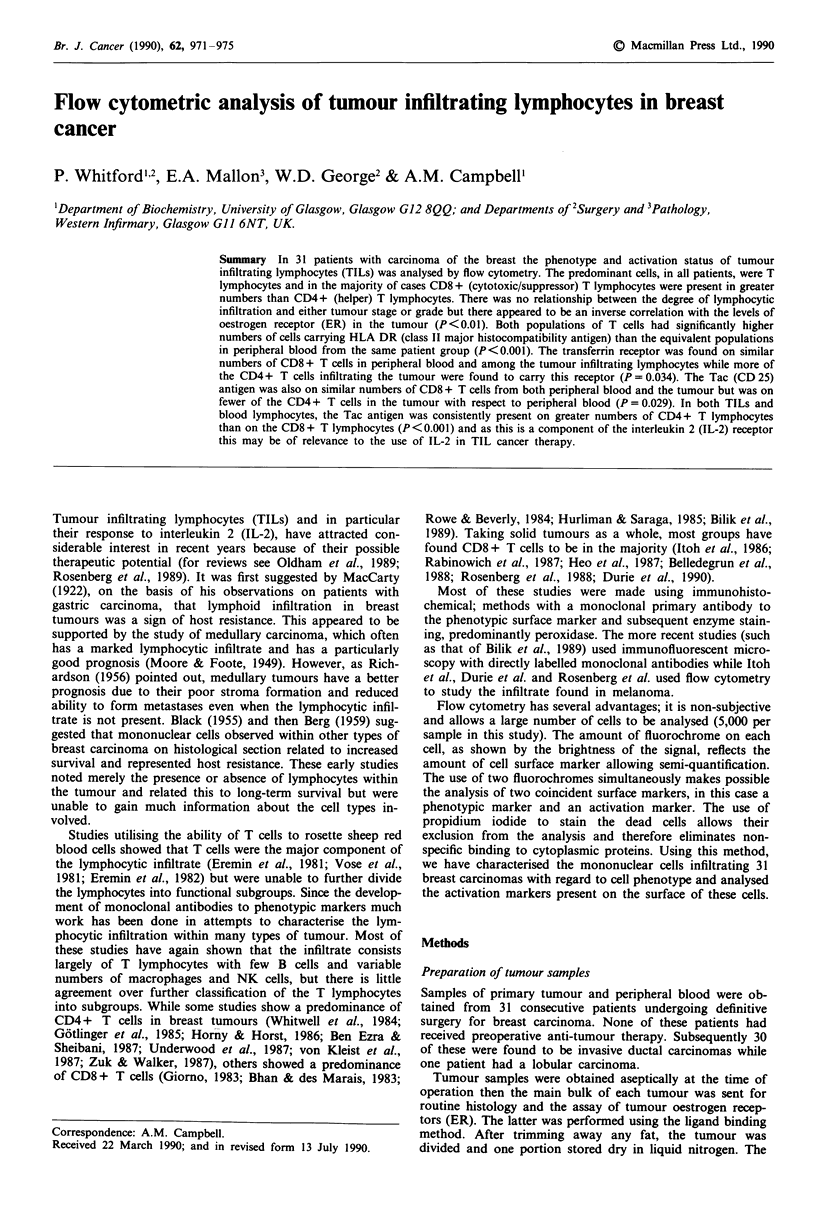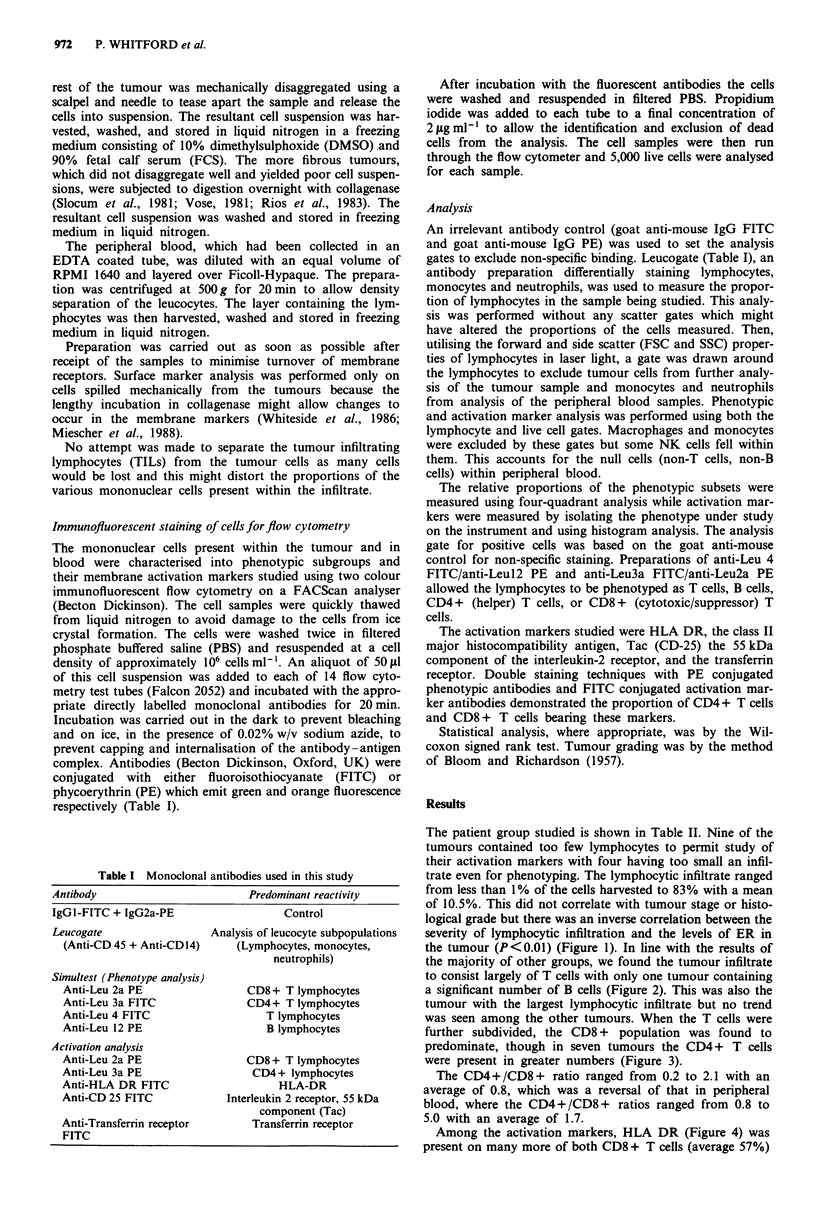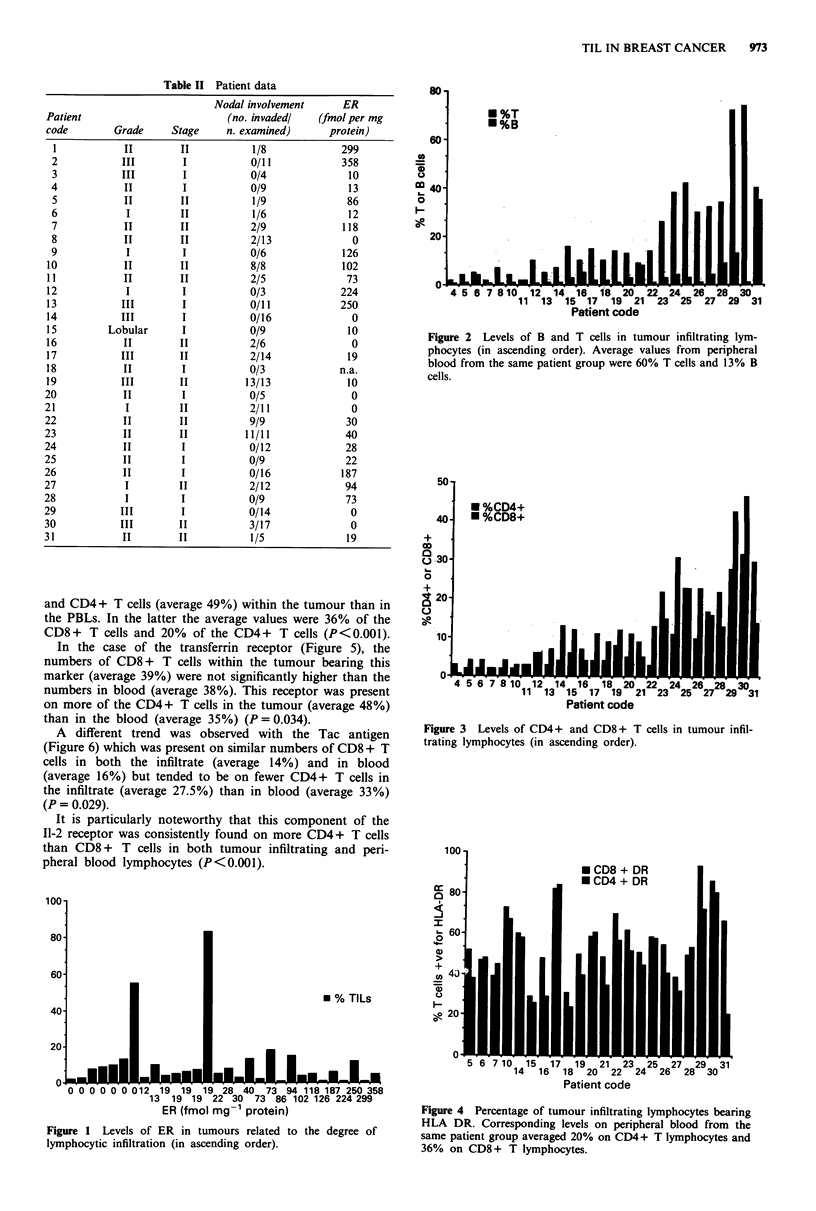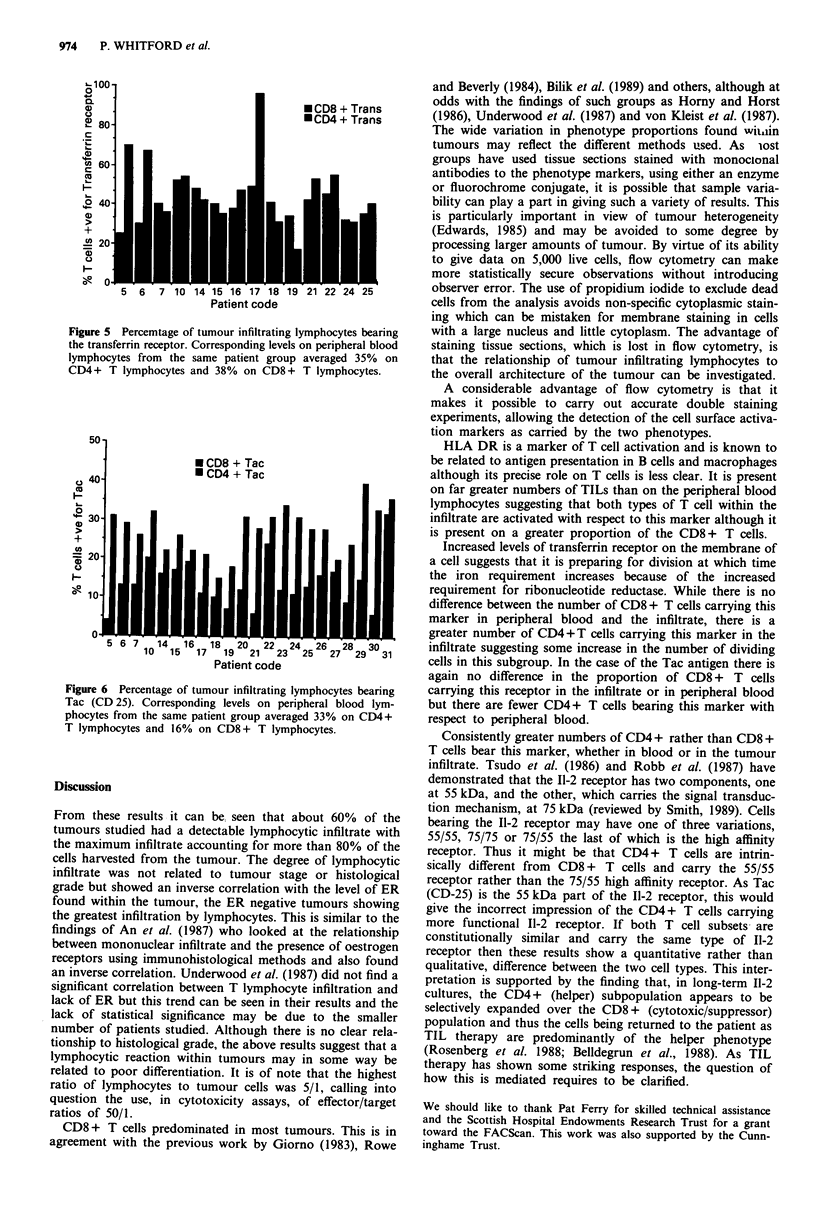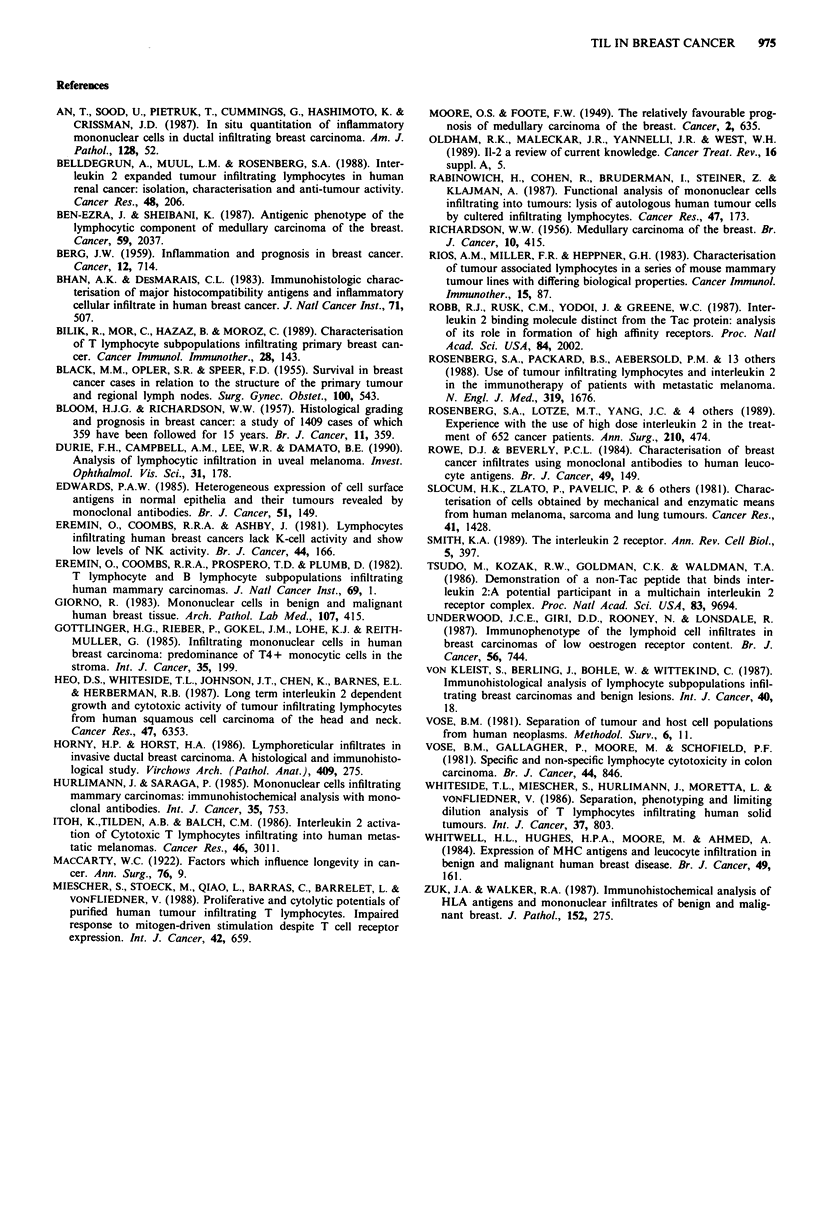# Flow cytometric analysis of tumour infiltrating lymphocytes in breast cancer.

**DOI:** 10.1038/bjc.1990.419

**Published:** 1990-12

**Authors:** P. Whitford, E. A. Mallon, W. D. George, A. M. Campbell

**Affiliations:** Department of Biochemistry, University of Glasgow, UK.

## Abstract

In 31 patients with carcinoma of the breast the phenotype and activation status of tumour infiltrating lymphocytes (TILs) was analysed by flow cytometry. The predominant cells, in all patients, were T lymphocytes and in the majority of cases CD8+ (cytotoxic/suppressor) T lymphocytes were present in greater numbers than CD4+ (helper) T lymphocytes. There was no relationship between the degree of lymphocytic infiltration and either tumour stage or grade but there appeared to be an inverse correlation with the levels of oestrogen receptor (ER) in the tumour (P less than 0.01). Both populations of T cells had significantly higher numbers of cells carrying HLA DR (class II major histocompatibility antigen) than the equivalent populations in peripheral blood from the same patient group (P less than 0.001). The transferrin receptor was found on similar numbers of CD8+ T cells in peripheral blood and among the tumour infiltrating lymphocytes while more of the CD4+ T cells infiltrating the tumour were found to carry this receptor (P = 0.034). The Tac (CD 25) antigen was also on similar numbers of CD8+ T cells from both peripheral blood and the tumour but was on fewer of the CD4+ T cells in the tumour with respect to peripheral blood (P = 0.029). In both TILs and blood lymphocytes, the Tac antigen was consistently present on greater numbers of CD4+ T lymphocytes than on the CD8+ T lymphocytes (P less than 0.001) and as this is a component of the interleukin 2 (IL-2) receptor this may be of relevance to the use of IL-2 in TIL cancer therapy.


Br. J. Cancer (1990), 62, 971-975 © Macmillan Press Ltd., 1990

### Flow cytometric analysis of tumour infiltrating lymphocytes in breast cancer

P. Whitford[1,2], E.A. Mallon[3], W.D. George[2] & A.M. Campbell[1]

[1]Department of Biochemistry, University of Glasgow, Glasgow G12 8QQ; and Departments of [2]Surgery and [3]Pathology, Western Infirmary, Glasgow G11 6NT, UK.

**Summary** In 31 patients with carcinoma of the breast the phenotype and activation status of tumour infiltrating lymphocytes (TILs) was analysed by flow cytometry. The predominant cells, in all patients, were T lymphocytes and in the majority of cases CD8+ (cytotoxic/suppressor) T lymphocytes were present in greater numbers than CD4+ (helper) T lymphocytes. There was no relationship between the degree of lymphocytic infiltration and either tumour stage or grade but there appeared to be an inverse correlation with the levels of oestrogen receptor (ER) in the tumour ($P<0.01$). Both populations of T cells had significantly higher numbers of cells carrying HLA DR (class II major histocompatibility antigen) than the equivalent populations in peripheral blood from the same patient group ($P<0.001$). The transferrin receptor was found on similar numbers of CD8+ T cells in peripheral blood and among the tumour infiltrating lymphocytes while more of the CD4+ T cells infiltrating the tumour were found to carry this receptor ($P=0.034$). The Tac (CD 25) antigen was also on similar numbers of CD8+ T cells from both peripheral blood and the tumour but was on fewer of the CD4+ T cells in the tumour with respect to peripheral blood ($P=0.029$). In both TILs and blood lymphocytes, the Tac antigen was consistently present on greater numbers of CD4+ T lymphocytes than on the CD8+ T lymphocytes ($P<0.001$) and as this is a component of the interleukin 2 (IL-2) receptor this may be of relevance to the use of IL-2 in TIL cancer therapy.

Tumour infiltrating lymphocytes (TILs) and in particular their response to interleukin 2 (IL-2), have attracted considerable interest in recent years because of their possible therapeutic potential (for reviews see Oldham *et al.*, 1989; Rosenberg *et al.*, 1989). It was first suggested by MacCarty (1922), on the basis of his observations on patients with gastric carcinoma, that lymphoid infiltration in breast tumours was a sign of host resistance. This appeared to be supported by the study of medullary carcinoma, which often has a marked lymphocytic infiltrate and has a particularly good prognosis (Moore & Foote, 1949). However, as Richardson (1956) pointed out, medullary tumours have a better prognosis due to their poor stroma formation and reduced ability to form metastases even when the lymphocytic infiltrate is not present. Black (1955) and then Berg (1959) suggested that mononuclear cells observed within other types of breast carcinoma on histological section related to increased survival and represented host resistance. These early studies noted merely the presence or absence of lymphocytes within the tumour and related this to long-term survival but were unable to gain much information about the cell types involved.

Studies utilising the ability of T cells to rosette sheep red blood cells showed that T cells were the major component of the lymphocytic infiltrate (Eremin *et al.*, 1981; Vose *et al.*, 1981; Eremin *et al.*, 1982) but were unable to further divide the lymphocytes into functional subgroups. Since the development of monoclonal antibodies to phenotypic markers much work has been done in attempts to characterise the lymphocytic infiltration within many types of tumour. Most of these studies have again shown that the infiltrate consists largely of T lymphocytes with few B cells and variable numbers of macrophages and NK cells, but there is little agreement over further classification of the T lymphocytes into subgroups. While some studies show a predominance of CD4+ T cells in breast tumours (Whitwell *et al.*, 1984; Götlinger *et al.*, 1985; Horny & Horst, 1986; Ben Ezra & Sheibani, 1987; Underwood *et al.*, 1987; von Kleist *et al.*, 1987; Zuk & Walker, 1987), others showed a predominance of CD8+ T cells (Giorno, 1983; Bhan & des Marais, 1983; Rowe & Beverly, 1984; Hurliman & Saraga, 1985; Bilik *et al.*, 1989). Taking solid tumours as a whole, most groups have found CD8+ T cells to be in the majority (Itoh *et al.*, 1986; Rabinowich *et al.*, 1987; Heo *et al.*, 1987; Belledegrun *et al.*, 1988; Rosenberg *et al.*, 1988; Durie *et al.*, 1990).

Most of these studies were made using immunohistochemical; methods with a monoclonal primary antibody to the phenotypic surface marker and subsequent enzyme staining, predominantly peroxidase. The more recent studies (such as that of Bilik *et al.*, 1989) used immunofluorescent microscopy with directly labelled monoclonal antibodies while Itoh *et al.*, Durie *et al.* and Rosenberg *et al.* used flow cytometry to study the infiltrate found in melanoma.

Flow cytometry has several advantages; it is non-subjective and allows a large number of cells to be analysed (5,000 per sample in this study). The amount of fluorochrome on each cell, as shown by the brightness of the signal, reflects the amount of cell surface marker allowing semi-quantification. The use of two fluorochromes simultaneously makes possible the analysis of two coincident surface markers, in this case a phenotypic marker and an activation marker. The use of propidium iodide to stain the dead cells allows their exclusion from the analysis and therefore eliminates non-specific binding to cytoplasmic proteins. Using this method, we have characterised the mononuclear cells infiltrating 31 breast carcinomas with regard to cell phenotype and analysed the activation markers present on the surface of these cells.

### Methods

### *Preparation of tumour samples*

Samples of primary tumour and peripheral blood were obtained from 31 consecutive patients undergoing definitive surgery for breast carcinoma. None of these patients had received preoperative anti-tumour therapy. Subsequently 30 of these were found to be invasive ductal carcinomas while one patient had a lobular carcinoma.

Tumour samples were obtained aseptically at the time of operation then the main bulk of each tumour was sent for routine histology and the assay of tumour oestrogen receptors (ER). The latter was performed using the ligand binding method. After trimming away any fat, the tumour was divided and one portion stored dry in liquid nitrogen. The

Correspondence: A.M. Campbell.
Received 22 March 1990; and in revised form 13 July 1990.

rest of the tumour was mechanically disaggregated using a scalpel and needle to tease apart the sample and release the cells into suspension. The resultant cell suspension was harvested, washed, and stored in liquid nitrogen in a freezing medium consisting of 10% dimethylsulphoxide (DMSO) and 90% fetal calf serum (FCS). The more fibrous tumours, which did not disaggregate well and yielded poor cell suspensions, were subjected to digestion overnight with collagenase (Slocum *et al.*, 1981; Vose, 1981; Rios *et al.*, 1983). The resultant cell suspension was washed and stored in freezing medium in liquid nitrogen.

The peripheral blood, which had been collected in an EDTA coated tube, was diluted with an equal volume of RPMI 1640 and layered over Ficoll-Hypaque. The preparation was centrifuged at 500 *g* for 20 min to allow density separation of the leucocytes. The layer containing the lymphocytes was then harvested, washed and stored in freezing medium in liquid nitrogen.

Preparation was carried out as soon as possible after receipt of the samples to minimise turnover of membrane receptors. Surface marker analysis was performed only on cells spilled mechanically from the tumours because the lengthy incubation in collagenase might allow changes to occur in the membrane markers (Whiteside *et al.*, 1986; Miescher *et al.*, 1988).

No attempt was made to separate the tumour infiltrating lymphocytes (TILs) from the tumour cells as many cells would be lost and this might distort the proportions of the various mononuclear cells present within the infiltrate.

### *Immunofluorescent staining of cells for flow cytometry*

The mononuclear cells present within the tumour and in blood were characterised into phenotypic subgroups and their membrane activation markers studied using two colour immunofluorescent flow cytometry on a FACScan analyser (Becton Dickinson). The cell samples were quickly thawed from liquid nitrogen to avoid damage to the cells from ice crystal formation. The cells were washed twice in filtered phosphate buffered saline (PBS) and resuspended at a cell density of approximately $10^6$ cells $ml^{-1}$. An aliquot of 50 $\mu l$ of this cell suspension was added to each of 14 flow cytometry test tubes (Falcon 2052) and incubated with the appropriate directly labelled monoclonal antibodies for 20 min. Incubation was carried out in the dark to prevent bleaching and on ice, in the presence of 0.02% w/v sodium azide, to prevent capping and internalisation of the antibody–antigen complex. Antibodies (Becton Dickinson, Oxford, UK) were conjugated with either fluoroisothiocyanate (FITC) or phycoerythrin (PE) which emit green and orange fluorescence respectively (Table I).

**Table I** Monoclonal antibodies used in this study

| *Antibody* | *Predominant reactivity* |
|---|---|
| IgG1-FITC + IgG2a-PE | Control |
| *Leucogate* (Anti-CD 45 + Anti-CD14) | Analysis of leucocyte subpopulations (Lymphocytes, monocytes, neutrophils) |
| *Simultest (Phenotype analysis)* | |
| Anti-Leu 2a PE | CD8+ T lymphocytes |
| Anti-Leu 3a FITC | CD4+ T lymphocytes |
| Anti-Leu 4 FITC | T lymphocytes |
| Anti-Leu 12 PE | B lymphocytes |
| *Activation analysis* | |
| Anti-Leu 2a PE | CD8+ T lymphocytes |
| Anti-Leu 3a PE | CD4+ lymphocytes |
| Anti-HLA DR FITC | HLA-DR |
| Anti-CD 25 FITC | Interleukin 2 receptor, 55 kDa component (Tac) |
| Anti-Transferrin receptor FITC | Transferrin receptor |

After incubation with the fluorescent antibodies the cells were washed and resuspended in filtered PBS. Propidium iodide was added to each tube to a final concentration of 2 $\mu g\ ml^{-1}$ to allow the identification and exclusion of dead cells from the analysis. The cell samples were then run through the flow cytometer and 5,000 live cells were analysed for each sample.

### *Analysis*

An irrelevant antibody control (goat anti-mouse IgG FITC and goat anti-mouse IgG PE) was used to set the analysis gates to exclude non-specific binding. Leucogate (Table I), an antibody preparation differentially staining lymphocytes, monocytes and neutrophils, was used to measure the proportion of lymphocytes in the sample being studied. This analysis was performed without any scatter gates which might have altered the proportions of the cells measured. Then, utilising the forward and side scatter (FSC and SSC) properties of lymphocytes in laser light, a gate was drawn around the lymphocytes to exclude tumour cells from further analysis of the tumour sample and monocytes and neutrophils from analysis of the peripheral blood samples. Phenotypic and activation marker analysis was performed using both the lymphocyte and live cell gates. Macrophages and monocytes were excluded by these gates but some NK cells fell within them. This accounts for the null cells (non-T cells, non-B cells) within peripheral blood.

The relative proportions of the phenotypic subsets were measured using four-quadrant analysis while activation markers were measured by isolating the phenotype under study on the instrument and using histogram analysis. The analysis gate for positive cells was based on the goat anti-mouse control for non-specific staining. Preparations of anti-Leu 4 FITC/anti-Leu12 PE and anti-Leu3a FITC/anti-Leu2a PE allowed the lymphocytes to be phenotyped as T cells, B cells, CD4+ (helper) T cells, or CD8+ (cytotoxic/suppressor) T cells.

The activation markers studied were HLA DR, the class II major histocompatibility antigen, Tac (CD-25) the 55 kDa component of the interleukin-2 receptor, and the transferrin receptor. Double staining techniques with PE conjugated phenotypic antibodies and FITC conjugated activation marker antibodies demonstrated the proportion of CD4+ T cells and CD8+ T cells bearing these markers.

Statistical analysis, where appropriate, was by the Wilcoxon signed rank test. Tumour grading was by the method of Bloom and Richardson (1957).

### Results

The patient group studied is shown in Table II. Nine of the tumours contained too few lymphocytes to permit study of their activation markers with four having too small an infiltrate even for phenotyping. The lymphocytic infiltrate ranged from less than 1% of the cells harvested to 83% with a mean of 10.5%. This did not correlate with tumour stage or histological grade but there was an inverse correlation between the severity of lymphocytic infiltration and the levels of ER in the tumour ($P < 0.01$) (Figure 1). In line with the results of the majority of other groups, we found the tumour infiltrate to consist largely of T cells with only one tumour containing a significant number of B cells (Figure 2). This was also the tumour with the largest lymphocytic infiltrate but no trend was seen among the other tumours. When the T cells were further subdivided, the CD8+ population was found to predominate, though in seven tumours the CD4+ T cells were present in greater numbers (Figure 3).

The CD4+/CD8+ ratio ranged from 0.2 to 2.1 with an average of 0.8, which was a reversal of that in peripheral blood, where the CD4+/CD8+ ratios ranged from 0.8 to 5.0 with an average of 1.7.

Among the activation markers, HLA DR (Figure 4) was present on many more of both CD8+ T cells (average 57%)

**Table II** Patient data

| *Patient code* | *Grade* | *Stage* | *Nodal involvement (no. invaded/ n. examined)* | *ER (fmol per mg protein)* |
|---|---|---|---|---|
| 1 | II | II | 1/8 | 299 |
| 2 | III | I | 0/11 | 358 |
| 3 | III | I | 0/4 | 10 |
| 4 | II | I | 0/9 | 13 |
| 5 | II | II | 1/9 | 86 |
| 6 | I | II | 1/6 | 12 |
| 7 | II | II | 2/9 | 118 |
| 8 | II | II | 2/13 | 0 |
| 9 | I | I | 0/6 | 126 |
| 10 | II | II | 8/8 | 102 |
| 11 | II | II | 2/5 | 73 |
| 12 | I | I | 0/3 | 224 |
| 13 | III | I | 0/11 | 250 |
| 14 | III | I | 0/16 | 0 |
| 15 | Lobular | I | 0/9 | 10 |
| 16 | II | II | 2/6 | 0 |
| 17 | III | II | 2/14 | 19 |
| 18 | II | I | 0/3 | n.a. |
| 19 | III | II | 13/13 | 10 |
| 20 | II | I | 0/5 | 0 |
| 21 | I | II | 2/11 | 0 |
| 22 | II | II | 9/9 | 30 |
| 23 | II | II | 11/11 | 40 |
| 24 | II | I | 0/12 | 28 |
| 25 | II | I | 0/9 | 22 |
| 26 | II | I | 0/16 | 187 |
| 27 | I | II | 2/12 | 94 |
| 28 | I | I | 0/9 | 73 |
| 29 | III | I | 0/14 | 0 |
| 30 | III | II | 3/17 | 0 |
| 31 | II | II | 1/5 | 19 |

and CD4+ T cells (average 49%) within the tumour than in the PBLs. In the latter the average values were 36% of the CD8+ T cells and 20% of the CD4+ T cells ($P<0.001$).

In the case of the transferrin receptor (Figure 5), the numbers of CD8+ T cells within the tumour bearing this marker (average 39%) were not significantly higher than the numbers in blood (average 38%). This receptor was present on more of the CD4+ T cells in the tumour (average 48%) than in the blood (average 35%) ($P=0.034$).

A different trend was observed with the Tac antigen (Figure 6) which was present on similar numbers of CD8+ T cells in both the infiltrate (average 14%) and in blood (average 16%) but tended to be on fewer CD4+ T cells in the infiltrate (average 27.5%) than in blood (average 33%) ($P=0.029$).

It is particularly noteworthy that this component of the Il-2 receptor was consistently found on more CD4+ T cells than CD8+ T cells in both tumour infiltrating and peripheral blood lymphocytes ($P<0.001$).

**Figure 1** Levels of ER in tumours related to the degree of lymphocytic infiltration (in ascending order).

**Figure 2** Levels of B and T cells in tumour infiltrating lymphocytes (in ascending order). Average values from peripheral blood from the same patient group were 60% T cells and 13% B cells.

**Figure 3** Levels of CD4+ and CD8+ T cells in tumour infiltrating lymphocytes (in ascending order).

**Figure 4** Percentage of tumour infiltrating lymphocytes bearing HLA DR. Corresponding levels on peripheral blood from the same patient group averaged 20% on CD4+ T lymphocytes and 36% on CD8+ T lymphocytes.

**Figure 5** Percemtage of tumour infiltrating lymphocytes bearing the transferrin receptor. Corresponding levels on peripheral blood lymphocytes from the same patient group averaged 35% on CD4+ T lymphocytes and 38% on CD8+ T lymphocytes.

**Figure 6** Percentage of tumour infiltrating lymphocytes bearing Tac (CD 25). Corresponding levels on peripheral blood lymphocytes from the same patient group averaged 33% on CD4+ T lymphocytes and 16% on CD8+ T lymphocytes.

### Discussion

From these results it can be seen that about 60% of the tumours studied had a detectable lymphocytic infiltrate with the maximum infiltrate accounting for more than 80% of the cells harvested from the tumour. The degree of lymphocytic infiltrate was not related to tumour stage or histological grade but showed an inverse correlation with the level of ER found within the tumour, the ER negative tumours showing the greatest infiltration by lymphocytes. This is similar to the findings of An *et al.* (1987) who looked at the relationship between mononuclear infiltrate and the presence of oestrogen receptors using immunohistological methods and also found an inverse correlation. Underwood *et al.* (1987) did not find a significant correlation between T lymphocyte infiltration and lack of ER but this trend can be seen in their results and the lack of statistical significance may be due to the smaller number of patients studied. Although there is no clear relationship to histological grade, the above results suggest that a lymphocytic reaction within tumours may in some way be related to poor differentiation. It is of note that the highest ratio of lymphocytes to tumour cells was 5/1, calling into question the use, in cytotoxicity assays, of effector/target ratios of 50/1.

CD8+ T cells predominated in most tumours. This is in agreement with the previous work by Giorno (1983), Rowe and Beverly (1984), Bilik *et al.* (1989) and others, although at odds with the findings of such groups as Horny and Horst (1986), Underwood *et al.* (1987) and von Kleist *et al.* (1987). The wide variation in phenotype proportions found within tumours may reflect the different methods used. As most groups have used tissue sections stained with monoclonal antibodies to the phenotype markers, using either an enzyme or fluorochrome conjugate, it is possible that sample variability can play a part in giving such a variety of results. This is particularly important in view of tumour heterogeneity (Edwards, 1985) and may be avoided to some degree by processing larger amounts of tumour. By virtue of its ability to give data on 5,000 live cells, flow cytometry can make more statistically secure observations without introducing observer error. The use of propidium iodide to exclude dead cells from the analysis avoids non-specific cytoplasmic staining which can be mistaken for membrane staining in cells with a large nucleus and little cytoplasm. The advantage of staining tissue sections, which is lost in flow cytometry, is that the relationship of tumour infiltrating lymphocytes to the overall architecture of the tumour can be investigated.

A considerable advantage of flow cytometry is that it makes it possible to carry out accurate double staining experiments, allowing the detection of the cell surface activation markers as carried by the two phenotypes.

HLA DR is a marker of T cell activation and is known to be related to antigen presentation in B cells and macrophages although its precise role on T cells is less clear. It is present on far greater numbers of TILs than on the peripheral blood lymphocytes suggesting that both types of T cell within the infiltrate are activated with respect to this marker although it is present on a greater proportion of the CD8+ T cells.

Increased levels of transferrin receptor on the membrane of a cell suggests that it is preparing for division at which time the iron requirement increases because of the increased requirement for ribonucleotide reductase. While there is no difference between the number of CD8+ T cells carrying this marker in peripheral blood and the infiltrate, there is a greater number of CD4+T cells carrying this marker in the infiltrate suggesting some increase in the number of dividing cells in this subgroup. In the case of the Tac antigen there is again no difference in the proportion of CD8+ T cells carrying this receptor in the infiltrate or in peripheral blood but there are fewer CD4+ T cells bearing this marker with respect to peripheral blood.

Consistently greater numbers of CD4+ rather than CD8+ T cells bear this marker, whether in blood or in the tumour infiltrate. Tsudo *et al.* (1986) and Robb *et al.* (1987) have demonstrated that the Il-2 receptor has two components, one at 55 kDa, and the other, which carries the signal transduction mechanism, at 75 kDa (reviewed by Smith, 1989). Cells bearing the Il-2 receptor may have one of three variations, 55/55, 75/75 or 75/55 the last of which is the high affinity receptor. Thus it might be that CD4+ T cells are intrinsically different from CD8+ T cells and carry the 55/55 receptor rather than the 75/55 high affinity receptor. As Tac (CD-25) is the 55 kDa part of the Il-2 receptor, this would give the incorrect impression of the CD4+ T cells carrying more functional Il-2 receptor. If both T cell subsets are constitutionally similar and carry the same type of Il-2 receptor then these results show a quantitative rather than qualitative, difference between the two cell types. This interpretation is supported by the finding that, in long-term Il-2 cultures, the CD4+ (helper) subpopulation appears to be selectively expanded over the CD8+ (cytotoxic/suppressor) population and thus the cells being returned to the patient as TIL therapy are predominantly of the helper phenotype (Rosenberg *et al.* 1988; Belldegrun *et al.*, 1988). As TIL therapy has shown some striking responses, the question of how this is mediated requires to be clarified.

We should like to thank Pat Ferry for skilled technical assistance and the Scottish Hospital Endowments Research Trust for a grant toward the FACScan. This work was also supported by the Cunninghame Trust.

### References

AN, T., SOOD, U., PIETRUK, T., CUMMINGS, G., HASHIMOTO, K. & CRISSMAN, J.D. (1987). In situ quantitation of inflammatory mononuclear cells in ductal infiltrating breast carcinoma. *Am. J. Pathol.*, **128**, 52.

BELLDEGRUN, A., MUUL, L.M. & ROSENBERG, S.A. (1988). Interleukin 2 expanded tumour infiltrating lymphocytes in human renal cancer: isolation, characterisation and anti-tumour activity. *Cancer Res.*, **48**, 206.

BEN-EZRA, J. & SHEIBANI, K. (1987). Antigenic phenotype of the lymphocytic component of medullary carcinoma of the breast. *Cancer*, **59**, 2037.

BERG, J.W. (1959). Inflammation and prognosis in breast cancer. *Cancer*, **12**, 714.

BHAN, A.K. & DESMARAIS, C.L. (1983). Immunohistologic characterisation of major histocompatibility antigens and inflammatory cellular infiltrate in human breast cancer. *J. Natl Cancer Inst.*, **71**, 507.

BILIK, R., MOR, C., HAZAZ, B. & MOROZ, C. (1989). Characterisation of T lymphocyte subpopulations infiltrating primary breast cancer. *Cancer Immunol. Immunother.*, **28**, 143.

BLACK, M.M., OPLER, S.R. & SPEER, F.D. (1955). Survival in breast cancer cases in relation to the structure of the primary tumour and regional lymph nodes. *Surg. Gynec. Obstet.*, **100**, 543.

BLOOM, H.J.G. & RICHARDSON, W.W. (1957). Histological grading and prognosis in breast cancer: a study of 1409 cases of which 359 have been followed for 15 years. *Br. J. Cancer*, **11**, 359.

DURIE, F.H., CAMPBELL, A.M., LEE, W.R. & DAMATO, B.E. (1990). Analysis of lymphocytic infiltration in uveal melanoma. *Invest. Ophthalmol. Vis. Sci.*, **31**, 178.

EDWARDS, P.A.W. (1985). Heterogeneous expression of cell surface antigens in normal epithelia and their tumours revealed by monoclonal antibodies. *Br. J. Cancer*, **51**, 149.

EREMIN, O., COOMBS, R.R.A. & ASHBY, J. (1981). Lymphocytes infiltrating human breast cancers lack K-cell activity and show low levels of NK activity. *Br. J. Cancer*, **44**, 166.

EREMIN, O., COOMBS, R.R.A., PROSPERO, T.D. & PLUMB, D. (1982). T lymphocyte and B lymphocyte subpopulations infiltrating human mammary carcinomas. *J. Natl Cancer Inst.*, **69**, 1.

GIORNO, R. (1983). Mononuclear cells in benign and malignant human breast tissue. *Arch. Pathol. Lab Med.*, **107**, 415.

GOTTLINGER, H.G., RIEBER, P., GOKEL, J.M., LOHE, K.J. & REITHMULLER, G. (1985). Infiltrating mononuclear cells in human breast carcinoma: predominance of T4+ monocytic cells in the stroma. *Int. J. Cancer*, **35**, 199.

HEO, D.S., WHITESIDE, T.L., JOHNSON, J.T., CHEN, K., BARNES, E.L. & HERBERMAN, R.B. (1987). Long term interleukin 2 dependent growth and cytotoxic activity of tumour infiltrating lymphocytes from human squamous cell carcinoma of the head and neck. *Cancer Res.*, **47**, 6353.

HORNY, H.P. & HORST, H.A. (1986). Lymphoreticular infiltrates in invasive ductal breast carcinoma. A histological and immunohistological study. *Virchows Arch. (Pathol. Anat.)*, **409**, 275.

HURLIMANN, J. & SARAGA, P. (1985). Mononuclear cells infiltrating mammary carcinomas: immunohistochemical analysis with monoclonal antibodies. *Int. J. Cancer*, **35**, 753.

ITOH, K.,TILDEN, A.B. & BALCH, C.M. (1986). Interleukin 2 activation of Cytotoxic T lymphocytes infiltrating into human metastatic melanomas. *Cancer Res.*, **46**, 3011.

MACCARTY, W.C. (1922). Factors which influence longevity in cancer. *Ann. Surg.*, **76**, 9.

MIESCHER, S., STOECK, M., QIAO, L., BARRAS, C., BARRELET, L. & VONFLIEDNER, V. (1988). Proliferative and cytolytic potentials of purified human tumour infiltrating T lymphocytes. Impaired response to mitogen-driven stimulation despite T cell receptor expression. *Int. J. Cancer*, **42**, 659.

MOORE, O.S. & FOOTE, F.W. (1949). The relatively favourable prognosis of medullary carcinoma of the breast. *Cancer*, **2**, 635.

OLDHAM, R.K., MALECKAR, J.R., YANNELLI, J.R. & WEST, W.H. (1989). Il-2 a review of current knowledge. *Cancer Treat. Rev.*, **16** suppl. A, 5.

RABINOWICH, H., COHEN, R., BRUDERMAN, I., STEINER, Z. & KLAJMAN, A. (1987). Functional analysis of mononuclear cells infiltrating into tumours: lysis of autologous human tumour cells by cultered infiltrating lymphocytes. *Cancer Res.*, **47**, 173.

RICHARDSON, W.W. (1956). Medullary carcinoma of the breast. *Br. J. Cancer*, **10**, 415.

RIOS, A.M., MILLER, F.R. & HEPPNER, G.H. (1983). Characterisation of tumour associated lymphocytes in a series of mouse mammary tumour lines with differing biological properties. *Cancer Immunol. Immunother.*, **15**, 87.

ROBB, R.J., RUSK, C.M., YODOI, J. & GREENE, W.C. (1987). Interleukin 2 binding molecule distinct from the Tac protein: analysis of its role in formation of high affinity receptors. *Proc. Natl Acad. Sci. USA*, **84**, 2002.

ROSENBERG, S.A., PACKARD, B.S., AEBERSOLD, P.M. & 13 others (1988). Use of tumour infiltrating lymphocytes and interleukin 2 in the immunotherapy of patients with metastatic melanoma. *N. Engl. J. Med.*, **319**, 1676.

ROSENBERG, S.A., LOTZE, M.T., YANG, J.C. & 4 others (1989). Experience with the use of high dose interleukin 2 in the treatment of 652 cancer patients. *Ann. Surg.*, **210**, 474.

ROWE, D.J. & BEVERLY, P.C.L. (1984). Characterisation of breast cancer infiltrates using monoclonal antibodies to human leucocyte antigens. *Br. J. Cancer*, **49**, 149.

SLOCUM, H.K., ZLATO, P., PAVELIC, P. & 6 others (1981). Characterisation of cells obtained by mechanical and enzymatic means from human melanoma, sarcoma and lung tumours. *Cancer Res.*, **41**, 1428.

SMITH, K.A. (1989). The interleukin 2 receptor. *Ann. Rev. Cell Biol.*, **5**, 397.

TSUDO, M., KOZAK, R.W., GOLDMAN, C.K. & WALDMAN, T.A. (1986). Demonstration of a non-Tac peptide that binds interleukin 2:A potential participant in a multichain interleukin 2 receptor complex. *Proc. Natl Acad. Sci. USA*, **83**, 9694.

UNDERWOOD, J.C.E., GIRI, D.D., ROONEY, N. & LONSDALE, R. (1987). Immunophenotype of the lymphoid cell infiltrates in breast carcinomas of low oestrogen receptor content. *Br. J. Cancer*, **56**, 744.

VON KLEIST, S., BERLING, J., BOHLE, W. & WITTEKIND, C. (1987). Immunohistological analysis of lymphocyte subpopulations infiltrating breast carcinomas and benign lesions. *Int. J. Cancer*, **40**, 18.

VOSE, B.M. (1981). Separation of tumour and host cell populations from human neoplasms. *Methodol. Surv.*, **6**, 11.

VOSE, B.M., GALLAGHER, P., MOORE, M. & SCHOFIELD, P.F. (1981). Specific and non-specific lymphocyte cytotoxicity in colon carcinoma. *Br. J. Cancer*, **44**, 846.

WHITESIDE, T.L., MIESCHER, S., HURLIMANN, J., MORETTA, L. & VONFLIEDNER, V. (1986). Separation, phenotyping and limiting dilution analysis of T lymphocytes infiltrating human solid tumours. *Int. J. Cancer*, **37**, 803.

WHITWELL, H.L., HUGHES, H.P.A., MOORE, M. & AHMED, A. (1984). Expression of MHC antigens and leucocyte infiltration in benign and malignant human breast disease. *Br. J. Cancer*, **49**, 161.

ZUK, J.A. & WALKER, R.A. (1987). Immunohistochemical analysis of HLA antigens and mononuclear infiltrates of benign and malignant breast. *J. Pathol.*, **152**, 275.